# Functional cure is associated with younger age in children undergoing antiviral treatment for active chronic hepatitis B

**DOI:** 10.1007/s12072-023-10631-9

**Published:** 2024-02-20

**Authors:** Min Zhang, Jing Li, Zhiqiang Xu, Peiyao Fan, Yi Dong, Fuchuan Wang, Yinjie Gao, Jianguo Yan, Lili Cao, Dong Ji, Danni Feng, Yanwei Zhong, Yang Zhang, Weiguo Hong, Chao Zhang, Fu-Sheng Wang

**Affiliations:** 1 Department of Liver Diseases, National Clinical Research Center for Infectious Diseases, The Fifth Medical Center of Chinese PLA General Hospital, Beijing, China; 2 Department of Infectious Diseases, National Clinical Research Center for Infectious Diseases, The Fifth Medical Center of Chinese PLA General Hospital, 100 Western 4th Ring Middle Road, Beijing, 100039 China; 3 302 Clinical Medical School, Peking University, Beijing, China

**Keywords:** Child, HBV, Chronic hepatitis B, Antiviral therapy, Interferons, Nucleoside Analogs, Functional cure, HBsAg loss, HBeAg seroconversion, HBV DNA loss

## Abstract

**Background and Aims:**

Functional cure is difficult to achieve using current antiviral therapies; moreover, limited data are available regarding treatment outcomes in children. This retrospective study aimed to assess the frequency of functional cure among children undergoing antiviral treatment for active chronic hepatitis B (CHB).

**Methods:**

A total of 372 children aged 1–16 years, with active CHB were enrolled and underwent either nucleos(t)ide analog monotherapy or combination therapy with interferon-α (IFN-α) for 24–36 months. All children attended follow-up visits every 3 months. Functional cure was defined as evidence of hepatitis B virus (HBV) DNA loss, circulating hepatitis B e antigen (HBeAg) loss/seroconversion, and hepatitis B surface antigen (HBsAg) loss.

**Results:**

After 36 months of antiviral treatment and/or follow-up visits, children with CHB aged 1– < 7 years exhibited higher rates of HBV DNA clearance, HBeAg seroconversion, and HBsAg loss than CHB children ≥ 7–16 years of age (93.75% versus [vs.] 86.21% [*p* < 0.0001]; 79.30% vs. 51.72% [*p* < 0.0001]; and 50.78% vs. 12.93% [*p* < 0.0001], respectively). Longitudinal investigation revealed more rapid dynamic reduction in HBV DNA, HBeAg, and HBsAg levels in children aged 1–7 years than in those aged ≥ 7–16 years with CHB. According to further age-stratified analysis, HBsAg loss rates were successively decreased in children with CHB who were 1– < 3, 3– < 7, 7– < 12, and 12–16 years of age (62.61% vs. 41.13% vs. 25.45% vs. 1.64%, respectively; *p* < 0.0001) at 36 months. In addition, baseline HBsAg level < 1,500 IU/mL was found to favor disease cure among these pediatric patients. No serious adverse events were observed throughout the study period.

**Conclusion:**

Results of the present study demonstrated that children aged 1– < 7 years, with active CHB can achieve a high functional cure rate by undergoing antiviral therapy compared to those aged ≥ 7 years, who undergo antiviral therapy. These data support the use of antiviral treatment at an early age in children with CHB. However, future prospectively randomized controlled trials are necessary to validate the findings of this study.

**Graphical Abstract:**

The younger age, the higher functional cure rate in children with chronic hepatitis B undergoing on-time antiviral treatment.
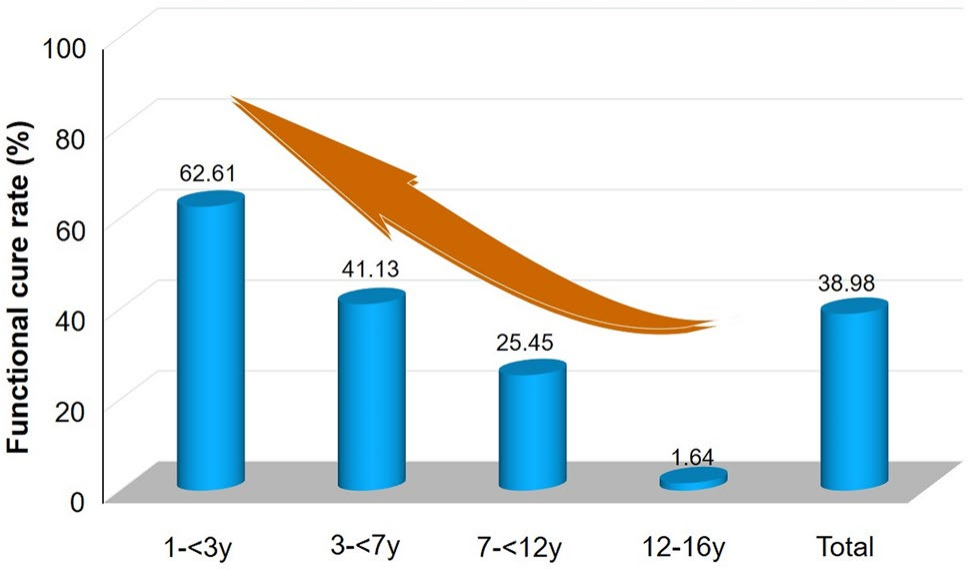

**Supplementary Information:**

The online version contains supplementary material available at 10.1007/s12072-023-10631-9.

## Introduction

Hepatitis B virus (HBV) infection remains a major threat to public health globally [1, 2]. Approximately 8–10% of newborns worldwide contract chronic hepatitis B (CHB) from HBV surface antigen (HBsAg)-positive mothers [3], with approximately 6 million children infected in China [4]. Children with hepatitis B e antigen (HBeAg)-positive CHB rarely spontaneously eradicate HBsAg without antiviral treatment, regardless of whether they have immune clearance CHB (IC-CHB) or immune tolerant CHB (IT-CHB). Active HBV replication in pediatric patients frequently generates significant quantities of virions, viral antigens, and covalently closed circular DNA (cccDNA). Additionally, viral DNA fragments can be readily integrated into the genomes of proliferative hepatocytes in these children. All of these factors combined may not only increase the risk for developing cirrhosis or hepatocellular carcinoma (HCC) but also lead to difficulties in curing the disease in adulthood [5, 6].

Functional cure for CHB is defined as the sustained loss of serum HBsAg and HBV DNA after a finite course of treatment [7]. Current antiviral drugs, including interferon-α (IFN-α) and nucleos(t)ide analogs (NAs), effectively suppress HBV replication and significantly minimize the risk for the disease progressing to cirrhosis and HCC [8, 9]. However, existing antiviral therapeutics, whether in isolation or in combination with IFN-α and NAs, rarely result in a functional cure for adult patients with CHB, although a small number of patients with a lower serum HBsAg load may be cured [7]. The IC phase is characterized by the presence of HBeAg, high serum HBV DNA and alanine aminotransferase (ALT) levels, and active inflammation and fibrosis in the liver [10]. Children in the periodic phase are recommended to undergo antiviral treatment according to guidelines or consensus. Different from adult patients, a substantial proportion of pediatric patients with CHB may be functionally cured using current antiviral treatment [9, 11–14]. Zhang et al. reported that combination therapy with IFN-α and NAs was more effective than IFN-α monotherapy in terms of viral suppression and serological responses in children with CHB [14]. Moreover, some studies have demonstrated that antiviral drugs, including IFN-α and/or NAs, are safe and well-tolerated in children [15, 16]. However, other studies have reported that antiviral treatment failed to improve functional cure, although current guidelines recommend initiating antiviral treatment in children with IC-CHB [17–19]. Explanations for these conflicting findings regarding the functional cure of pediatric CHB remain unclear. To address this knowledge gap, the present real-world study analyzed 372 children with IC-CHB who underwent 2–3 years of IFN-based antiviral treatment to evaluate the functional cure rate and potential factors influencing this outcome.

## Methods and materials

### Study population

**Fig. 1:**
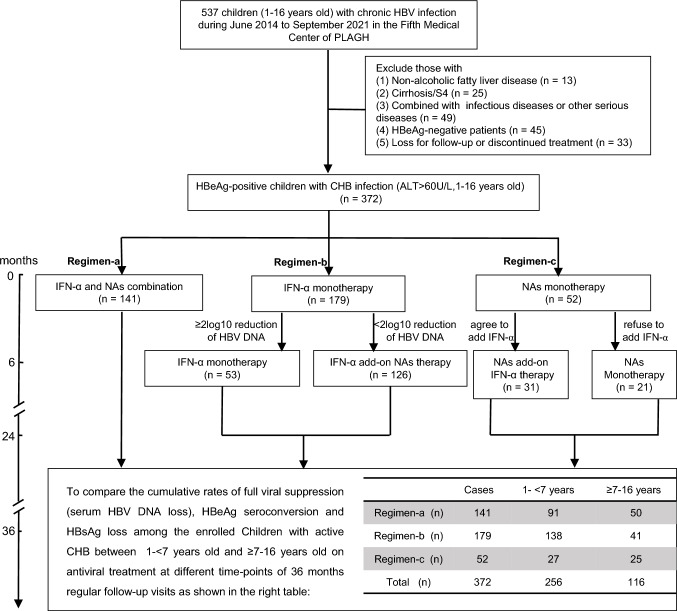
Flow diagram of the retrospective study. Of 537 children with CHB aged 1–16 years, a total of 372 were deemed eligible according to the inclusion and exclusion criteria and enrolled for antiviral treatment under the following regimens: Regimen-a comprised IFN-α treatment plus NAs over the course of 24 months; Regimen-b consisted of initial treatment with IFN-α monotherapy for six months and an additional treatment of 18 months with IFN-α monotherapy in cases of a ≥ 2 log_10_ decrease in serum HBV DNA; otherwise, the extensional treatment used IFN-α add-on lamivudine (3 mg/kg/day) or entecavir (0.015 mg/kg/day); Regimen-c, for those initially reluctant to undergo IFN-α treatment, involved NAs monotherapy over 36 months. Nevertheless, some individuals exhibited a poor viral response to NAs monotherapy after six months and shifted to an NAs add-on IFN-α treatment plan after obtaining their guardians’ consent. All patients received antiviral treatment and follow-up visits during the 36-month research period. The study also divided the 372 cases by age group for further analysis of their cumulative rates of HBV DNA loss, HBeAg seroconversion, and HBsAg loss. Abbreviations: *HBV* hepatitis B virus, *CHB* chronic hepatitis B, *IFN-α* interferon-α, *NAs* nucleos(t)ide analogs

This retrospective study included 537 consecutive children 1–16 years of age, who were diagnosed with CHB and admitted to our unit between June 2014 and September 2021. Of these 537 children, 165 were excluded due to non-alcoholic fatty liver disease (n = 13), cirrhosis (n = 25), infectious or other serious diseases (n = 49), HBeAg-negative CHB (n = 45), lack of follow-up, or discontinued treatment (n = 33). The remaining 372 children, who were HBeAg-positive with IC-CHB, underwent antiviral therapy and were regularly followed up every 3 months over a 36-month period, were included (Fig. 1). The inclusion criteria were as follows [9]: HBsAg positivity ≥ 6 months; serum HBV DNA > 2 × 10^4^ IU/mL; persistent serum HBeAg positivity; serum ALT levels fulfilling the diagnostic criteria (> 60 U/L) for 2 consecutive tests within a period of at least 3–6 months; and written informed consent from the child’s parent/guardian. Individuals with liver cirrhosis (meta-analysis of histological data in viral hepatitis [i.e., “Metavir”] F4 or equivalent) [20] or HCC, patients who underwent antiviral treatment for chronic HBV infection, those with co-infection with hepatitis A, C, D, or E, those with Epstein-Barr virus, cytomegalovirus, or human immunodeficiency virus, a history of mental disorders, and those with other serious co-existing diseases, such as severe thyroid disease and retinopathy, were excluded.

### Antiviral regimens and follow-up visits

#### Antiviral regimens

As illustrated in Fig. 1, this study used three different antiviral regimens (a, b, and c) to empirically treat children with IC-CHB. Of the 372 children who participated in the study, Regimen-a included 141 children, each of whom underwent IFN-α treatment in combination with NAs. Regimen-b included 179 children, each of whom underwent IFN-α monotherapy. After 6 months, the antiviral regimens were modified in accordance with previous studies [9]. In Regimen-b, 53 of the 179 children who exhibited a ≥ 2 log_10_ decrease in serum HBV DNA (according to real-time quantitative polymerase chain reaction, with a cut-off value of 40 IU/mL) after 6 months therapy continued IFN-α monotherapy for a further 18 months, with a follow-up visit at 36 months. Moreover, 126 of these 179 children exhibited a < 2 log_10_ decrease of serum HBV DNA after 6 months of therapy; thus, for these patients, lamivudine (LAM) or entecavir (ETV) was added. Regimen-c included the remaining 52 children with IC-CHB, all of whom were reluctant to undergo IFN-α treatment at baseline and were administered NAs monotherapy. Of these, 21 completed the 36-month course, while 31 underwent combined treatment involving NAs and IFN-α after 6 months, either because their guardian had consented to the addition of IFN-α in the first instance or did so later because the child subsequently exhibited a poor viral response to NAs monotherapy. All children underwent antiviral treatment with follow-up visits throughout the 36-month study period.

In these treatment regimens, IFN-α was generally administered for a finite period of 24 months, whereas NAs were used for up to 36 months and then reviewed; treatment was continued or discontinued according to viral response of the children. Because the guidelines encourage antiviral therapy for children with IC-CHB [19, 21], this study did not include untreated children in the control group.

#### Antiviral drugs

For IFN-α treatment, IFN-α was administered to children with CHB aged 1–16 years, by subcutaneous injection in single doses of 3 MU/m^2^ on alternate days over the course of 1 week. This was then increased to 6 MU/m^2^ (up to 10 MU/m^2^) on alternate days, for the remaining administration period. For NAs treatment, LAM (3 mg/kg/day) was administered to children aged ≤ 2 years [17] and ETV (0.015 mg/kg/day) to those aged > 2 years [21]. Additionally, the efficacy of antiviral therapy was evaluated in accordance with a previous study [22].

### Safety evaluation

Safety evaluations included the incidence of adverse events, laboratory investigations, growth parameters, and vital signs. Safety was assessed at each follow-up visit during the 36-month study period. Laboratory investigations included standard hematology, coagulation function, blood chemistry, and thyroid function.

### Serological assays

Routine blood tests, biochemical liver function assessments, plasma HBV DNA quantification, and serological HBV marker evaluations were performed at baseline and at each follow-up visit every 3 months according to our previous study [22]. Peripheral lymphocytes and related subsets were analyzed as previously described [23]. HBV genotypes were determined by phylogenetic analysis of the 1225-base pair *S/Pol* gene sequence (nt 54–1278), as described previously [24].

### Histological analysis

Liver histology using a one-second needle biopsy was performed in 329 of the 372 children with CHB before antiviral treatment, in accordance with a standard protocol [25]. Liver tissue samples were fixed, embedded, stained, and scored by a senior pathologist in a blinded manner. Histological analysis of liver tissues was performed to assess fibrosis stage and the grade of inflammatory activity, in accordance with a previous study [13].

### Statistical analysis

Baseline parameters for all participants were analyzed using the chi-squared or Fisher’s exact tests for categorical variables and the Mann–Whitney U test for quantitative variables [26]. Factors influencing HBsAg loss were investigated using the Cox proportional hazard model [27]. The cumulative rates of HBsAg loss, HBeAg seroconversion, and HBV DNA loss were calculated using the Kaplan–Meier method and compared using log-rank tests. A propensity score matching (PSM) analysis was subsequently implemented to avoid baseline parameter bias that could influence the efficacy of antivirus treatment [28]. Propensity scores were derived using multiple logistic regression analysis and matched for participants in the two age groups (i.e., 1 to < 7 and ≥ 7–16 years) (1:1) using the nearest-neighbor matching algorithm with a standard deviation of 0.05 from the estimated propensity scores. All statistical analyses were performed using R version 4.1.1 (R Foundation for Statistical Computing, Vienna, Austria; http://www.r-project.org/). Differences with a two-tailed *p* < 0.05 were considered to be statistically significant.

## Results

### Baseline characteristics of children with CHB and factors involved in functional cure

The present cohort comprised 372 children with IC-CHB, who were positive for HBeAg and admitted to our unit between June 2014 and September 2021, including 239 boys and 133 girls, with a median age of 4.60 years (interquartile range [IQR] 2.68–8.22 years). Baseline demographic, virological, immunological, and liver histological parameters of the children, as well as their treatment responses at the end of the 36-month study period are summarized in Table 1. The cases were predominately HBV genotype C infections (252/372 [67.7%]), with only 61 children with genotype B (16.4%). The median baseline levels of serum HBsAg, HBV DNA, and ALT were 4.09 log_10_ IU/mL (IQR 3.64–4.48 log_10_ IU/mL), 7.65 log_10_ IU/mL (IQR 7.00–8.23 log_10_ IU/mL), and 120.00 U/L (IQR 83.97–212.00 U/L), respectively. Histological liver examination revealed that 228 children exhibited inflammation grade ≥ 2 (228/329 [69.3%]), and nearly one-half exhibited stage ≥ 2 fibrosis (156/329 [47.4%]).

**Table 1:** Baseline characteristics and clinical outcomes of the three antiviral regimen groups, and the risk factors involved in hepatitis B surface antigen loss

	Total cases					Univariate COX analysis		Multivariate COX analysis	
Characteristics	Overall	Regimen-a	Regimen-b	Regimen-c	*p* value	HR (95% CI)	*p* value	HR (95% CI)	*p* value
N	372	141	179	52	–	–	–	–	–
Age, years	4.60 [2.68, 8.22]	5.20 [3.20, 9.50]	3.70 [2.30, 6.35]	6.45 [2.03, 13.18]	0.001	0.778 (0.730, 0.828)	< 0.001	0.714 (0.633, 0.806)	< 0.001
Sex					0.208				
Male	239 (64.2)	93 (66.0)	108 (60.4)	38 (73.1)		1		1	
Female	133 (35.8)	48 (34.0)	71 (39.7)	14 (26.9)		1.768 (1.274, 2.453)	0.001	1.767 (1.126, 2.774)	0.013
qHBsAg, log_10_IU/mL	4.09 [3.64, 4.48]	4.20 [3.74, 4.52]	4.09 [3.60, 4.49]	3.81 [3.47, 4.15]	0.011	0.798 (0.652, 0.976)	0.028	0.493 (0.38, 0.639)	< 0.001
HBV DNA, log_10_IU/mL	7.65 [7.00, 8.23]	7.82 [7.26, 8.39]	7.54 [6.77, 8.16]	7.29 [7.00, 8.06]	0.003	0.878 (0.756, 1.020)	0.089		
ALT, U/L	120.00 [83.97, 212.00]	121.00 [87.00, 210.00]	111.00 [76.50, 185.50]	177.50 [99.75,371.50]	0.001	0.999 (0.997, 1)	0.046	1.001 (0.999, 1.002)	0.410
Neutrophils count, 10^9^/L	2.40 [1.80, 3.13]	2.28 [1.80, 3.10]	2.50 [1.85, 3.12]	2.23 [1.64, 3.20]	0.412	1.134 (1.004, 1.280)	0.042	1.151 (0.98, 1.352)	0.087
CD4 + T cells count, cells/μL	1273.00 [905.50,1823.50]	1124.00 [840.25, 1550.50]	1379.00 [953.00,1862.00]	1060.00 [804.00,2120.00]	0.040	11.845 (4.755, 29.501)	< 0.001	0.433 (0.079, 2.384)	0.336
CD8 + T cells count, cells/μL	856.00 [672.00,1198.25]	837.50 [714.50, 1178.50]	902.00 [654.00, 1201.00]	803.00 [680.00,1201.00]	0.675	6.074 (2.315, 15.940)	< 0.001	1.496 (0.344, 6.513)	0.591
B cells count, cells/μL	787.00 [483.25,1235.50]	712.50 [474.75, 999.75]	903.00 [574.00, 1262.00]	642.00 [389.00,1350.00]	0.043	7.883 (3.773, 16.466)	< 0.001	0.790 (0.219, 2.851)	0.718
NK cells count, cells/μL	221.00 [149.25,389.75]	218.50 [150.50, 361.50]	224.00 [146.00, 445.00]	221.00 [158.00, 323.00]	0.535	3.042 (1.740, 5.316)	< 0.001	1.476 (0.749, 2.907)	0.260
HBV genotype, n (%)					0.027				
B genotype	61 (16.4)	22 (15.6)	33 (18.4)	6 (11.5)		1			
C genotype	252 (67.7)	94 (66.7)	127 (70.9)	31 (59.6)		0.991 (0.644, 1.525)	0.968		
Undetected	59 (15.9)	25 (17.7)	19 (10.6)	15 (28.8)		0.633 (0.344, 1.167)	0.143		
Histological evaluation									
Grade of inflammation^a^, *n* (%)					0.013				
< 2	101 (30.7)	36 (29.3)	58 (36.7)	7 (14.6)		1			
≥ 2	228 (69.3)	87 (70.7)	100 (63.3)	41 (85.4)		0.56 (0.396, 0.792)	0.001		
Stage of fibrosis^a^, *n* (%)					0.064				
< 2	173 (52.6)	62 (50.4)	92 (58.2)	19 (39.6)		1			
≥ 2	156 (47.4)	61 (49.6)	66 (41.8)	29 (60.4)		0.514 (0.339, 0.777)	0.002		
Clinical outcome, *n* (%)									
HBV DNA loss	340 (91.4)	128 (90.8)	162 (90.5)	50 (96.2)	0.418				
HBeAg seroconversion	263 (70.7)	96 (68.1)	131 (73.2)	36 (69.2)	0.591				
HBsAg loss	145 (39.0)	54 (38.3)	75 (41.9)	16 (30.8)	0.343				

^a^Liver histological analyses were performed in 329/372 children before treatment. Categorical variables expressed as number (*n*) and percentage (%), and continuous variables as median [interquartile range]. The clinical outcome was evaluated at 36 months of this study

*HBV* hepatitis B virus, *ALT* alanine aminotransferase, *qHBsAg* quantitative hepatitis B surface antigen, *HBeAg* hepatitis B e antigen, *NK* natural killer, *HR* hazard ratio, *CI* confidence interval

In general, ideal treatment responses to antiviral therapy begin with HBV DNA loss, followed by HBeAg loss with seroconversion, and HBsAg loss. The cumulative rates of serum HBV DNA loss, HBeAg seroconversion, and HBsAg loss in the 372 children with IC-CHB after 36 months were 91.4% (340/372), 70.7% (263/372), and 39.0% (145/372), respectively (Table 1). No differences were present in the cumulative rates of serum HBV DNA loss (*p* = 0.418), HBeAg seroconversion (*p* = 0.591), or HBsAg loss (*p* = 0.343) among the three antiviral regimens after the study (Table 1). Nevertheless, multivariate Cox regression analysis indicated that baseline age was an important independent factor influencing HBsAg loss (Table 1), whereas both sex and baseline quantitative HBsAg were found to influence HBsAg loss.

Because there were no significant differences in antiviral responses across the different regimens (Table 1), the 372 children were divided into 2 age groups―group-A (1– < 7 years [n = 256]) and group-B (≥ 7–16 years [n = 116]), to compare the potential impact of age on functional cure in the children with IC-CHB undergoing antiviral therapy [29]. As described in the Statistical analysis section, PSM was performed at a 1:1 ratio to adjust for any potential bias in the comparison between younger and older children. After PSM, no significant differences were observed in sex composition, serum baseline HBsAg, ALT levels, inflammation grade, fibrosis stage, or, in particular, the antiviral regimen adopted between the 1– < 7 years (PSM-group-A [n = 79]) and 7–16 (PSM-group-B [n = 79]) years age groups (Table 2).

**Table 2:** Baseline characteristics and clinical outcome according to the two-level age grouping

	Total-cases			PSM-cases (1:1 pair)		
Characteristics	group-A	group-B	*p* value	PSM-group-A	PSM-group-B	*p* value
N	256	116	–	79	79	–
Age, years	3.20 [1.90, 4.80]	12.20 [9.02, 14.00]	< 0.001	3.50 [2.45, 4.90]	12.20 [8.60, 13.90]	< 0.001
Female, *n* (%)	105 (41.0)	28 (24.1)	0.002	22 (27.8)	21 (26.6)	1
qHBsAg, log_10_IU/mL	4.23 [3.64, 4.54]	3.95 [3.63, 4.23]	0.001	3.86 [3.49, 4.26]	3.94 [3.52, 4.22]	0.574
HBV DNA, log_10_IU/mL	7.65 [7.00, 8.24]	7.62 [7.00, 8.18]	0.392	7.36 [6.88, 8.02]	7.54 [6.81, 8.19]	0.625
ALT, U/L	110.00 [80.75, 181.25]	168.00 [92.25, 252.75]	< 0.001	112.00 [86.00, 186.00]	152.00 [86.50, 234.00]	0.083
Neutrophils count, 10^9^/L	2.44 [1.79, 3.25]	2.32 [1.84, 3.01]	0.351	2.44 [1.75, 3.10]	2.32 [1.88, 2.98]	0.709
CD4 + T cells count, cells/μL	1527.00 [1113.00, 1971.50]	853.00 [773.00, 1075.50]	< 0.001	1288.00 [172.50, 1820.50]	813.00 [589.00, 1046.00]	0.001
CD8 + T cells count, cells/μL	969.00 [774.00, 1350.00]	724.00 [566.50, 914.50]	< 0.001	774.00 [180.50, 1112.50]	635.00 [482.00, 844.50]	0.070
B cells count, cells/μL	952.00 [695.50, 1383.00]	435.00 [349.50, 628.50]	< 0.001	730.00 [94.00, 1207.00]	410.00 [281.50, 613.00]	< 0.001
NK cells count, cells/μL	235.00 [158.50, 475.00]	176.00 [129.50, 278.50]	< 0.001	183.00 [29.50, 276.50]	166.00 [96.00, 270.50]	0.778
HBV genotype, *n* (%)			0.220			0.769
B genotype	44 (17.2)	17 (14.7)		12 (15.2)	15 (19.0)	
C genotype	177 (69.1)	75 (64.7)		57 (72.2)	53 (67.1)	
Undetected	35 (13.7)	24 (20.7)		10 (12.7)	11 (13.9)	
Histological evaluation						
Grade of inflammation^a^, *n* (%)			< 0.001			0.434
G1	83 (36.1)	18 (18.2)		23 (29.1)	17 (21.5)	
G2	131 (57.0)	59 (59.6)		47 (59.5)	49 (62.0)	
G3	16 (7.0)	22 (22.2)		9 (11.4)	13 (16.5)	
Stage of fibrosis^a^, *n* (%)			0.008			0.973
S0	1 (0.4)	0 (0.0)		0 (0.0)	0 (0.0)	
S1	133 (57.8)	39 (39.4)		34 (43.0)	34 (43.0)	
S2	73 (31.7)	40 (40.4)		32 (40.5)	33 (41.8)	
S3	23 (10.0)	20 (20.2)		13 (16.5)	12 (15.2)	
Treatment, *n* (%)			0.001			0.335
Regimen-a	91 (35.6)	50 (43.1)		41 (51.9)	32 (40.5)	
Regimen-b	138 (53.9)	41 (35.3)		28 (35.4)	33 (41.8)	
Regimen-c	27 (10.5)	25 (21.6)		10 (12.7)	14 (17.7)	
Clinical outcome, *n* (%)						
HBV DNA loss	240 (93.8)	100 (86.2)	0.028	75 (94.9)	66 (83.5)	0.040
HBeAg seroconversion	203 (79.3)	60 (51.7)	< 0.001	65 (82.3)	39 (49.4)	< 0.001
HBsAg loss	130 (50.8)	15 (12.9)	< 0.001	41 (51.9)	11 (13.9)	< 0.001

^a^Liver histological analyses were performed in 329/372 children before treatment. Categorical variables expressed as number (*n*) and percentage (%), and continuous variables as median [interquartile range]. The clinical outcome was evaluated at 36 months of this study

*PSM* propensity score matching, *group-A* aged 1– < 7 years without PSM, *group-B* aged ≥ 7–16 years without PSM, *PSM group-A* aged 1– < 7 years with PSM, *PSM group-B* aged ≥ 7–16 years with PSM, *HBV* hepatitis B virus, *ALT* alanine aminotransferase, *qHBsAg* quantitative hepatitis B surface antigen, *HBeAg* hepatitis B e antigen, *NK* natural killer

## Faster and more cumulative viral suppression in younger children with IC-CHB undergoing antiviral therapy

**Fig. 2:**
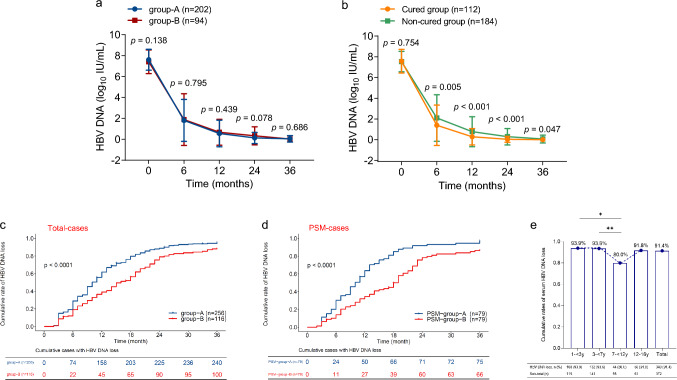
Dynamic changes of serum HBV DNA levels and the cumulative rate of HBV DNA loss in children with IC-CHB during the 36-month study period. **a** Serum HBV DNA changes in the children with CHB in group-A (1– < 7-year-olds) and group-B (≥ 7–16-.\year-olds); **b** Serum HBV DNA changes in the cured and non-cured children; **c**–**d** the cumulative rates of serum HBV DNA loss in different age groups; **c** the total number of cases; **d** PSM cases; and **e** comparisons of serum HBV DNA loss in the different age groups at the end of the 36-month follow-up period. Seventy-six patients lacked HBV DNA data, as they had received a follow-up by telephone call rather than visiting our hospital in this retrospective study. There were 11 children with serum positive HBV DNA in the 7– < 12 age-group, but nine of 11 cases had low loads of HBV DNA on antiviral treatment. For example, seven of 11 children were with HBV DNA loads ranging within 50–100 IU/mL and two of 11 children were with positive HBV DNA loads ranging within 100–200 IU/mL. Abbreviations: *: *p* < 0.05; **: *p* < 0.01; *HBV* hepatitis B virus, *CHB* chronic hepatitis B, *IC* immune clearance, *PSM* propensity score matching

Analysis revealed that serum HBV DNA levels plummeted within the first 6 months of treatment in group-A and group-B (Fig. 2a) and, by the end of the 12th month, had fallen to approximately normal levels (Fig. 2a). By 24 months, serum HBV DNA levels in group-A were lower than those in group-B, although the difference was marginally outside the threshold for significance (*p* = 0.078). After classifying the 372 children as either cured or non-cured, further analysis revealed that cured children exhibited lower HBV DNA levels than the non-cured after 6 months (Fig. 2b). Faster and deeper inhibition of HBV replication was found in cured than in non-cured participants, especially within the first 12 months. Notably, although children in the non-cured group did not achieve functional cure at the end of the study, HBV DNA levels in the majority of HBV DNA-positive children ranged from 40 to 200 IU/mL at the 36-month point (Fig. 2b).

A two-age stratification analysis of the 372 children with CHB revealed that there was a significantly higher rate of HBV DNA loss in group-A than in group-B at 6, 12, 24, and 36 months after the initiation of the antiviral treatment: 28.9 vs. 19.0%; 61.7 vs. 38.8%; 87.9 vs. 77.6%; and 93.8 vs. 86.2%, respectively (Fig. 2c). In other words, serum HBV DNA loss occurred earlier in group-A than in group-B. Similar observations were made in the PSM groups (Fig. 2d). Of the three regimens, Regimen-c yielded a faster and earlier antiviral response in terms of HBV DNA loss than Regimen-b or Regimen-a (Supplementary Fig. 1a). The 1– < 7 years subgroup in each regimen demonstrated a higher rate of cumulative HBV DNA loss at the 36-month follow-up visit, with *p* = 0.053 for Regimen-a, *p* < 0.001 for Regimen-b, and *p* = 0.063 for Regimen-c (Supplementary Fig. 1b–d) compared with the ≥ 7–16-years subgroup. Furthermore, stratifying the age variable into four groups (1– < 3, 3– < 7, 7– < 12, and 12–16 years) (Supplementary Fig. 2) produced results similar to those of the two age-group analyses, which exhibited rapid HBV DNA loss within 24 months of baseline. As shown in Fig. 2e, at 36 months, the 2 younger groups (i.e., 1– < 3 and 3– < 7 years) exhibited higher rates of HBV DNA loss (93.9 and 93.6%) than the two older groups (80.0 and 91.8%). Notably, the lower HBV DNA loss rate in the 7– < 12 years age group may be associated with the small sample size (n = 55) compared with the other three groups; a high proportion of children underwent IFN-α monotherapy (6/55 [10.9%]) and the antiviral efficacy of IFN-α therapy in controlling viral replication was limited compared with NAs.

Previous studies have confirmed that elevated serum ALT level at baseline is a predisposing factor for HBeAg seroconversion in children with CHB [30]. In the current cohort, ALT levels dropped rapidly within the first 6 months of antiviral treatment in both groups (Supplementary Fig. 3a). The dynamic changes in serum ALT levels that were characteristic of group-A (i.e., 1– < 7 years of age) contrasted with those in group-B (i.e., ≥ 7–16 years of age) (*p* = 0.033, Supplementary Fig. 3a). In group-A, there were no differences in serum ALT levels in the cured or non-cured subgroups (Supplementary Fig. 3b). However, baseline serum ALT levels and serum ALT (*p* = 0.029) declined in the non-cured group-B subgroup, exceeding those of the cured subgroups, especially within 6 months of starting treatment (Supplementary Fig. 3c).

## Higher cumulative HBeAg seroconversion rates in younger children undergoing antiviral therapy for IC-CHB

**Fig. 3:**
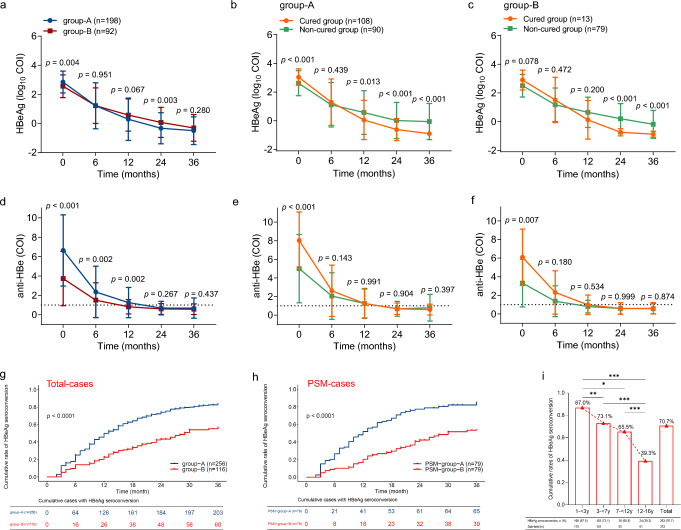
Dynamic changes of serum HBeAg and anti-HBe levels and cumulative rates of HBeAg seroconversion in children with IC-CHB during the 36-month study period. **a** Serum HBeAg changes in the children with CHB in group-A (1– < 7-year-olds) and group-B (≥ 7–16-year-olds); **b**–**c** Serum HBeAg changes in the cured and non-cured children in the two groups; **d** Serum anti-HBe changes in the children with CHB in the two groups; **e**–**f** Serum anti-HBe changes in the cured and non-cured children in the two groups; and **g**–**h** the cumulative rates of HBeAg seroconversion in the different age groups. **g** The total number of cases; **h** total number of PSM cases; and **i** a comparison of the HBeAg seroconversion rates in the different age groups at the end of the 36-month follow-up period. Eighty-two patients lacked HBeAg and anti-HBe data, as they had received a follow-up by telephone rather than visiting our hospital in this retrospective study. Abbreviations: *PSM* propensity score matching, *: *p* < 0.05; **: *p* < 0.01; ***: *p* < 0.001; *IC-CHB* immune clearance chronic hepatitis B, *HBeAg* hepatitis B e antigen

Next, dynamic changes in HBeAg loss and seroconversion throughout the study period were analyzed. Baseline HBeAg level in group-A was significantly higher than that in group-B (*p* = 0.004) (Fig. 3a); however, by 24 months, group-A had decreased significantly (*p* = 0.003). Similarly, as demonstrated in Fig. 3b and c, HBeAg level in the cured subgroup among those in group-A was significantly higher at the outset of treatment, although by 12 months, it had fallen to a significantly lower level than that observed in the non-cured subgroup (*p* = 0.013, Fig. 3b). Furthermore, HBeAg level did not significantly vary between the cured and non-cured subgroups in group-B until 24 months, when the level was significantly lower in the cured than that of the non-cured subgroup (*p* < 0.001, Fig. 3c).

A decrease in the cut-off index (COI) value for anti-HBe to < 1 indicated that it had become positive. Within the first 12 months, the decrease in anti-HBe in group-A was significantly greater than that in group-B (Fig. 3d) and, by 24 months, the median COI value for anti-HBe dropped to < 1 in both group-A and group-B, with no difference between the cured and non-cured subgroups (Fig. 3e, f). These results indicate that 24 months of antiviral therapy in children with IC-CHB can lead to HBeAg seroconversion.

The cumulative HBeAg seroconversion rate was exceedingly high in all 372 children with CHB at 36 months (263/372 [70.7%]) (Table 1). An analysis based on the two-age stratification revealed that the group-A exhibited 1.81 times the cumulative rate of HBeAg seroconversion than group-B at the 6-month mark (25.0 vs. 13.8%; *p* = 0.014). Such a significant difference between the 2 groups was consistently observed at 12, 24, and 36 months: 49.2 vs. 22.4%, 71.9 vs. 41.4%, and 79.3 vs. 51.7%, respectively (Fig. 3g). Moreover, age effects were observed in the PSM processing data, with a *p* < 0.001 (Fig. 3h). Comparing the cumulative HBeAg seroconversion rates of the children under the 3 regimens, those who underwent treatment with Regimen-c achieved HBeAg seroconversion sooner than those treated with other regimens (Supplementary Fig. 4a). Similarly, younger cases under each antiviral regimen exhibited higher cumulative rates of HBeAg seroconversion at the end of the study, with *p* values as follows: Regimen-a, *p* < 0.001; Regimen-b, *p* < 0.001; and Regimen-c, *p* = 0.036 (Supplementary Fig. 4b–d). Collectively, these findings demonstrate that children aged 1– < 7 years had higher cumulative HBeAg seroconversion rates than those aged ≥ 7–16 years.

The cumulative rate of HBeAg seroconversion decreased in inverse proportion to age in the four age groups, irrespective of when the follow-up analysis occurred, both without (Supplementary Fig. 5a) and with (Supplementary Fig. 5b) PSM processing. In the final follow-up of the children without PSM processing, the cumulative HBeAg seroconversion rate decreased as baseline age increased, with 87.0% in the 1– < 3, 73.1% in the 3– < 7, 65.5% in the 7– < 12 and, finally, 39.3% in the 12–16 years age groups (Fig. 3i), which is still greater than (or near to) that reported in cases of adult CHB [31].

## Higher functional cure rate in younger children undergoing antiviral therapy for IC-CHB

**Fig. 4:**
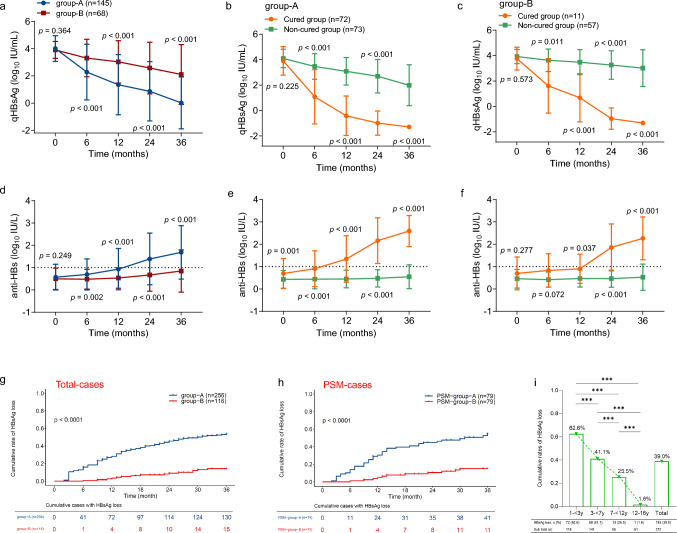
Dynamic changes of serum HBsAg and anti-HBs levels and cumulative rates of HBsAg loss in children with IC-CHB during the 36-month study period. **a** Serum HBsAg changes in the children with CHB in group-A (1– < 7-year-olds) and group-B (≥ 7–16-year-olds); **b**–**c** Serum HBsAg changes in the cured and non-cured children in the two groups; **d** Serum anti-HBs changes of the children in the two groups during the antiviral treatment period; **e**–**f** Serum anti-HBs changes in the cured and non-cured children in the two groups; **g**–**h** cumulative rates of serum HBsAg loss in the different age groups; **g** the total number of cases; **h** PSM cases; and **i** a comparison of HBsAg loss in the different age groups at the end of the 36-month follow-up period. A total of 159 patients lacked HBsAg and anti-HBs data, as they had received a follow-up by telephone rather than visiting our hospital in this retrospective study. Abbreviations: *PSM* propensity score matching. ***: *p* < 0.001; *IC-CHB* immune clearance chronic hepatitis B, *HBeAg* hepatitis B e antigen

As shown in Fig. 4a, serum HBsAg levels continuously decreased after treatment was initiated in both group-A and group-B. Nevertheless, the reduction in serum HBsAg levels in group-A was more pronounced and occurred significantly faster than that in group-B (Fig. 4a). Regardless of whether group-A or group-B, the reduction in serum HBsAg levels in the cured subgroup was faster than that in the non-cured subgroup (Fig. 4b, c). Accordingly, serum anti-HBs levels continuously and quickly increased after the initial treatment in group-A compared with group-B (Fig. 4d), and by 6 months, they had far outstripped the level in group-B (*p* = 0.002). In contrast to group-A, in which anti-HBs levels in the cured subgroups were significantly higher than those in the non-cured subgroup at each time point (Fig. 4e), group-B exhibited no significant difference in anti-HBs levels of the cured and non-cured groups until 12 months (Fig. 4f).

By the end of the study, the cumulative HBsAg loss rate was 39.0% (145/372) among 372 children with IC-CHB. Of the 256 children in group-A, 130 (50.8%) were functionally cured, whereas only 15 of 116 (12.9%) in group-B achieved HBsAg loss (*p* < 0.001) (Fig. 4g, Table 2). In accordance with the results of this real-world cohort, those in PSM-group-A achieved a higher HBsAg loss rate than those in PSM-group-B during antiviral therapy (*p* < 0.001), as illustrated in Fig. 4h. The observation that younger children aged 1– < 7 years had a higher functional cure rate than those aged ≥ 7–16 years was also found in each antiviral regimen group, with the following *p* values: Regimen-a, *p* < 0.001; Regimen-b, *p* < 0.001; and Regimen-c, *p* = 0.002 (Supplementary Fig. 6).

Four-age group stratification analysis of the 372 participants further revealed that the cumulative HBsAg loss rate increased as treatment progressed (Supplementary Fig. 7). Taking the last time point as an example, as baseline age increased, the rate of cumulative HBsAg loss quickly decreased to 62.6% in the 1 to < 3, 41.1% in the 3– < 7, 25.5% in the 7– < 12, and 1.6% in the 12–16 years age groups (Fig. 4i).

Previous studies have confirmed that a high HBsAg level is an important hurdle in attempts to achieve cure for HBV infection [7, 30]. Data reported in Table 1 demonstrate that baseline HBsAg level was associated with functional cure, which was consistent with both univariate and multivariate Cox regression analyses. A statistical analysis based on the two HBsAg levels revealed that the baseline HBsAg < 1500 IU/mL had a higher cumulative HBsAg loss than HBsAg ≥ 1500 IU/mL at each follow-up (*p* = 0.002, Supplementary Fig. 8a). In both the HBsAg < 1500 IU/mL and HBsAg ≥ 1500 IU/mL groups, the younger children (< 7 years) exhibited higher cumulative HBsAg loss rates than the older children (≥ 7 years) (Supplementary Fig. 8b).

### Safety evaluation

No serious adverse events were observed in any of the 372 children with CHB who underwent antiviral treatment during the 36-month follow-up period. However, mild to moderate adverse events occurred in 351 children who underwent IFN-α therapy. The two most common adverse events observed during IFN-α treatment were fever (219/351) and neutropenia (203/351) (Table 3), followed by fatigue (114/351), nausea (112/351), and alopecia (81/351). Other adverse events included thyroid dysfunction (27/351), arthralgia (17/351), rashes (15/351), thrombocytopenia (13/351), and growth retardation (5/351). All of these adverse events or symptoms spontaneously resolved or reversed once IFN-α treatment was discontinued, consistent with previous studies [32, 33]. Essentially, children with CHB demonstrated acceptable safety and tolerability to antiviral therapy.

**Table 3:** Cumulative adverse events occurring throughout antiviral treatment and follow-up period

Types	Cases	Treatment	Outcome
Fever	219 (62.4%)	Acetaminophen or without treatment	Relief for spontaneous remission
Neutropenia	203 (57.8%)	Without treatment	Relief after discontinuation of IFN-α therapy
Fatigue	114 (32.5%)	Without treatment	Self-relief
Nausea	112 (31.9%)	Without treatment	Self-relief
Alopecia	81 (23.1%)	Without treatment	Self-relief
Thyroid dysfunction	27 (7.7%)	Without treatment	Relief after discontinuation of IFN-α therapy
Arthralgia	17 (4.8%)	Without treatment	Self-relief
Rash	15 (4.3%)	Without treatment	Self-relief
Thrombocytopenia	13 (3.7%)	Without treatment	Relief after discontinuation of IFN-α therapy
Growth retardation	5 (1.4%)	Without treatment	Growth returned to normal after cessation of therapy

## Discussion

To achieve a functional cure for CHB, enormous effort has been invested in clinical trials to explore novel antiviral drugs and immunomodulatory approaches to reduce HBsAg levels and restore impaired HBV-specific immune responses. However, there have been no breakthroughs in significantly improving the functional cure of CHB in adult patients. Our study found that antiviral treatment for 24–36 months was safe in children with IC-CHB, with clinical cure rates of up to 39.0% (145/372). Although children in the IC phase may undergo spontaneous HBeAg clearance after a follow-up period of several months or years, HBsAg loss rarely occurs without antiviral treatment. The results also indicated that baseline age at antiviral treatment may be a critical factor influencing the functional cure of CHB.

When a functional cure is achieved, sequential HBV DNA loss, HBeAg seroconversion, and HBsAg loss often occur. By the end of this study, the various age groups had achieved a cumulative HBV DNA loss rate of up to approximately 90%, with no substantial differences among the different age groups. However, compared with older children, HBV DNA replication was rapidly inhibited in younger children on initiation of antiviral treatment. This may, in part, explain why the cumulative rates of HBeAg seroconversion and HBsAg loss were generally higher in younger than in older children at the 24- and 36-month follow-up visits. In fact, after 12 months of antiviral therapy, many non-cured cases had already occurred or approached HBeAg seroconversion and HBV DNA loss despite detectable HBsAg. This state is an intermediate step toward an HBV cure, also known as a partial HBV cure [7, 34]. For non-cured or older children, an appropriate extension of the antiviral therapy period is necessary. Throughout the follow-up visits, only two among the 145 cured children became HBsAg-positive; however, they retained HBV DNA negativity, HBeAg seroconversion, and normalization of ALT levels.

Previous studies have indicated that multiple factors, such as antiviral drugs, sex, and baseline levels of HBV DNA, HBeAg, and HBsAg, are likely to influence the efficacy of antiviral treatment [14]. Antiviral regimen(s), sex, and baseline serum HBsAg levels appeared to play much less of a role in promoting a functional cure in children undergoing antiviral therapy for IC-CHB compared with baseline age. Early implementation of antiviral treatment in younger children with IC-CHB may be a feasible approach to cure this disease.

Host HBV-specific adaptive immune responses can potentially influence the functional cure for CHB [7]. In this study, univariate regression analysis revealed that baseline levels of CD4-positive ( +) T cells (*p* < 0.001), CD8^+^ T cells (*p* < 0.001), natural killer (NK) cells (*p* < 0.001), B cells (*p* < 0.001), and neutrophils (*p* = 0.042) were significantly associated with functional cure (Table 1). This result is similar to that of a previous study [35]. Accordingly, baseline CD4^+^ T, CD8^+^ T, and B cell counts decreased as age increased in the present study (Supplementary Fig. 9). As mentioned above, high baseline levels of these three immune cell populations were positively associated with functional cure. However, the underlying mechanism requires further investigation.

Here, we attempted to develop a nomogram prediction model for the prognosis of children with IC-CHB using baseline age and other independent factors influencing HBsAg loss, including sex and baseline HBsAg levels. The nomogram calibration curve for predicting the probability of HBsAg loss at 36 months after the commencement of antiviral treatment, based on a training cohort of 372 cases, demonstrated a reasonable level of accuracy in predicting the probability of HBsAg loss (Supplementary Fig. 10). In summary, our prognostic nomogram model for children undergoing antiviral therapy for CHB provides preliminary evidence that age-dependent characteristics may have important clinical applications in achieving a functional cure.

Nevertheless, the present study had several limitations, the first of which was the inclusion of only patients with the hepatis B/C genotype. Second, multiple complex antiviral regimens were adopted, each of which was highly effective in bringing about a functional cure in children with IC-CHB; thus, it was difficult to determine which antiviral regimen was best. Third, the sample size of the 7– < 12-year age group was relatively small, and a small number of cases were followed up by telephone and lacked serological data. In addition, the follow-up period was relatively short. Finally, this study did not explore the mechanism underlying the effect of age on antiviral therapy in children with IC-CHB.

In conclusion, the current study found that children undergoing antiviral therapy for IC-CHB exhibited a high functional cure rate, and that younger age was positively associated with functional cure rate. These findings provide preliminary evidence for decision-making regarding antiviral treatment in children with IC-CHB. However, a prospective, multicenter, large-sample clinical trial is necessary to confirm the safety and efficacy of antiviral therapy in children with this condition. In addition, the related viral and immune mechanisms underlying the higher functional cure rate must be addressed in future studies.

### Supplementary Information

Below is the link to the electronic supplementary material.

Supplementary file1 (PPTX 2054 KB)

## Data Availability

All data supporting the findings in this study are available from the corresponding author upon reasonable request.
