# Supplementary material for: Functional cure is associated with younger age in children undergoing antiviral treatment for active chronic hepatitis B
Source: Hepatol Int. 2024 Feb 20;18(2):435–48. doi: 10.1007/s12072-023-10631-9 (PMC11014810; doi:10.1007/s12072-023-10631-9)
Supplement: Supplementary file 1 — Supplementary file1 (PPTX 2054 KB) [file 12072_2023_10631_MOESM1_ESM.pptx]

## Slide 1
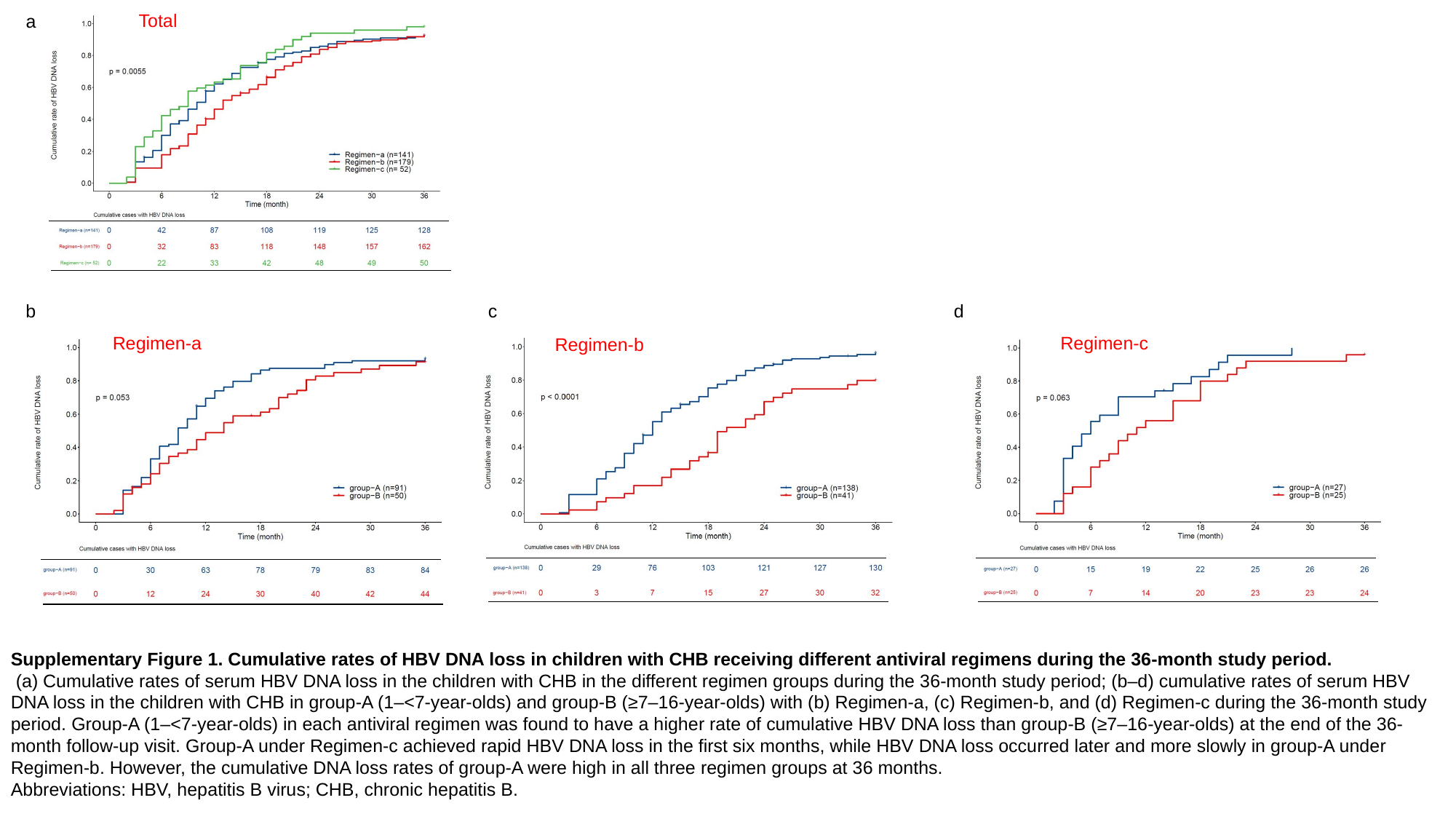

Total
a
d
b
c
Regimen-a
Regimen-c
Regimen-b
Supplementary Figure 1. Cumulative rates of HBV DNA loss in children with CHB receiving different antiviral regimens during the 36-month study period.
 (a) Cumulative rates of serum HBV DNA loss in the children with CHB in the different regimen groups during the 36-month study period; (b–d) cumulative rates of serum HBV DNA loss in the children with CHB in group-A (1–<7-year-olds) and group-B (≥7–16-year-olds) with (b) Regimen-a, (c) Regimen-b, and (d) Regimen-c during the 36-month study period. Group-A (1–<7-year-olds) in each antiviral regimen was found to have a higher rate of cumulative HBV DNA loss than group-B (≥7–16-year-olds) at the end of the 36-month follow-up visit. Group-A under Regimen-c achieved rapid HBV DNA loss in the first six months, while HBV DNA loss occurred later and more slowly in group-A under Regimen-b. However, the cumulative DNA loss rates of group-A were high in all three regimen groups at 36 months.
Abbreviations: HBV, hepatitis B virus; CHB, chronic hepatitis B.

## Slide 2
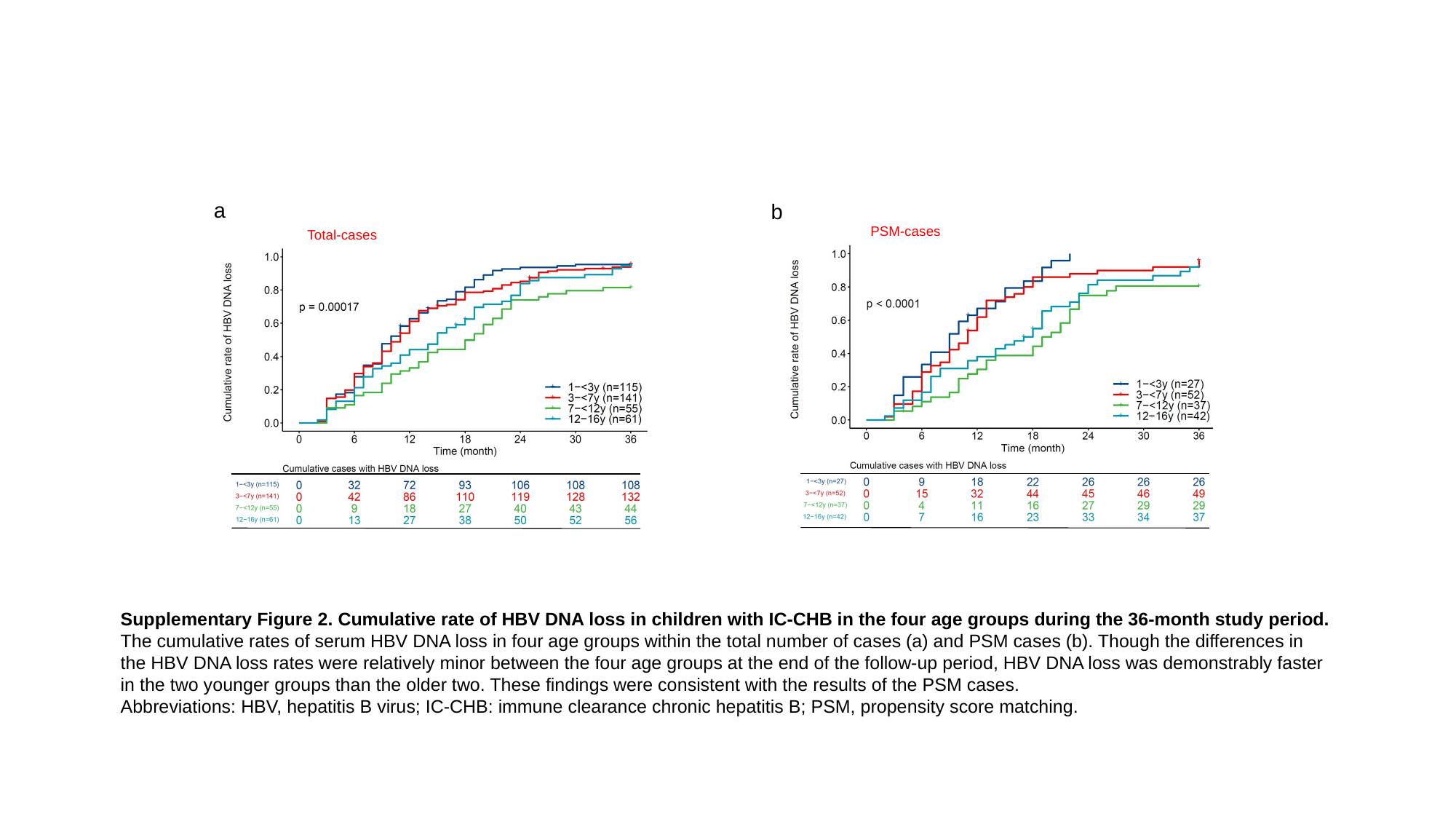

a
b
PSM-cases
Total-cases
Supplementary Figure 2. Cumulative rate of HBV DNA loss in children with IC-CHB in the four age groups during the 36-month study period.
The cumulative rates of serum HBV DNA loss in four age groups within the total number of cases (a) and PSM cases (b). Though the differences in the HBV DNA loss rates were relatively minor between the four age groups at the end of the follow-up period, HBV DNA loss was demonstrably faster in the two younger groups than the older two. These findings were consistent with the results of the PSM cases.
Abbreviations: HBV, hepatitis B virus; IC-CHB: immune clearance chronic hepatitis B; PSM, propensity score matching.

## Slide 3
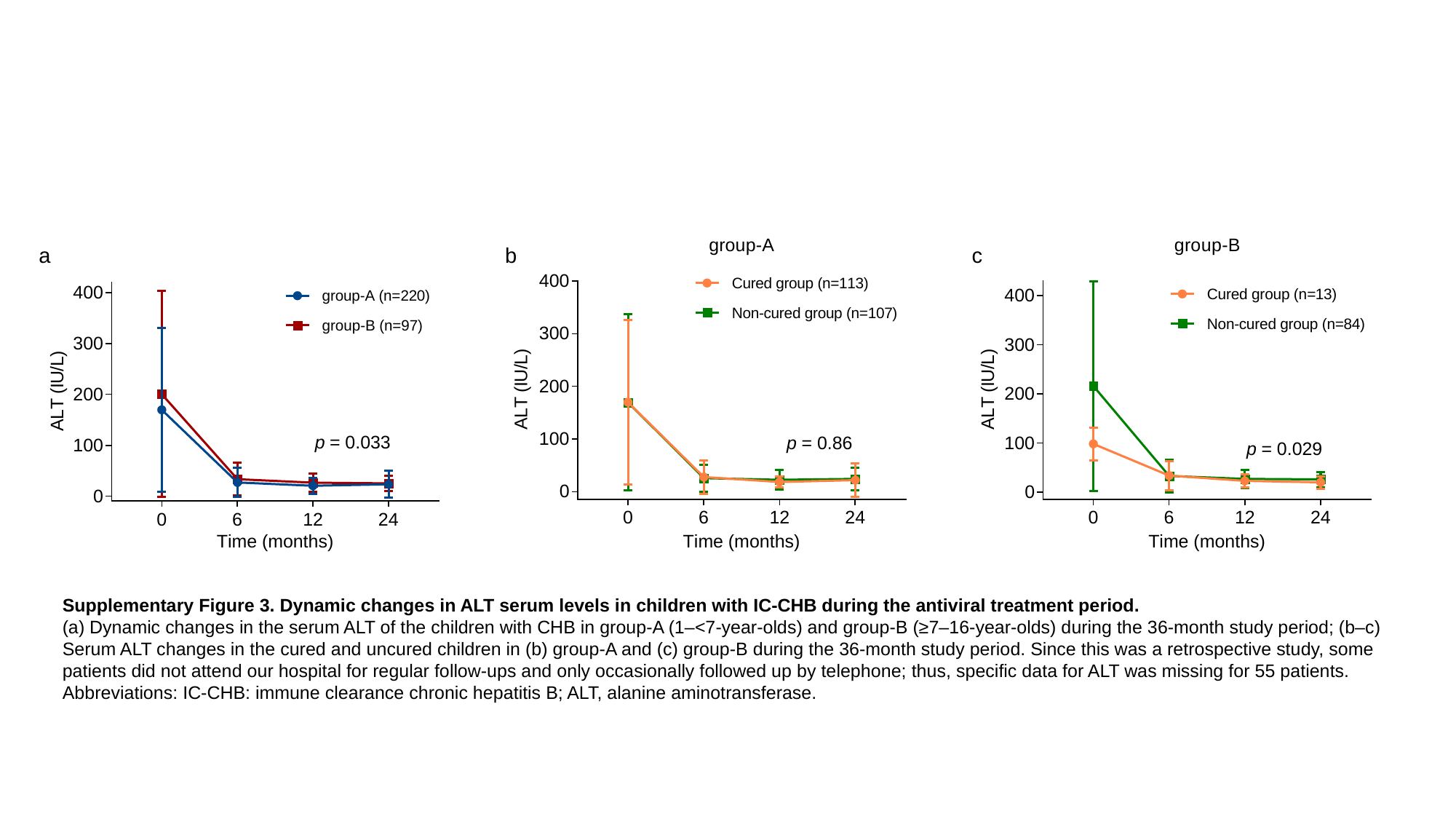

b
c
a
Supplementary Figure 3. Dynamic changes in ALT serum levels in children with IC-CHB during the antiviral treatment period.
(a) Dynamic changes in the serum ALT of the children with CHB in group-A (1–<7-year-olds) and group-B (≥7–16-year-olds) during the 36-month study period; (b–c) Serum ALT changes in the cured and uncured children in (b) group-A and (c) group-B during the 36-month study period. Since this was a retrospective study, some patients did not attend our hospital for regular follow-ups and only occasionally followed up by telephone; thus, specific data for ALT was missing for 55 patients.
Abbreviations: IC-CHB: immune clearance chronic hepatitis B; ALT, alanine aminotransferase.

## Slide 4
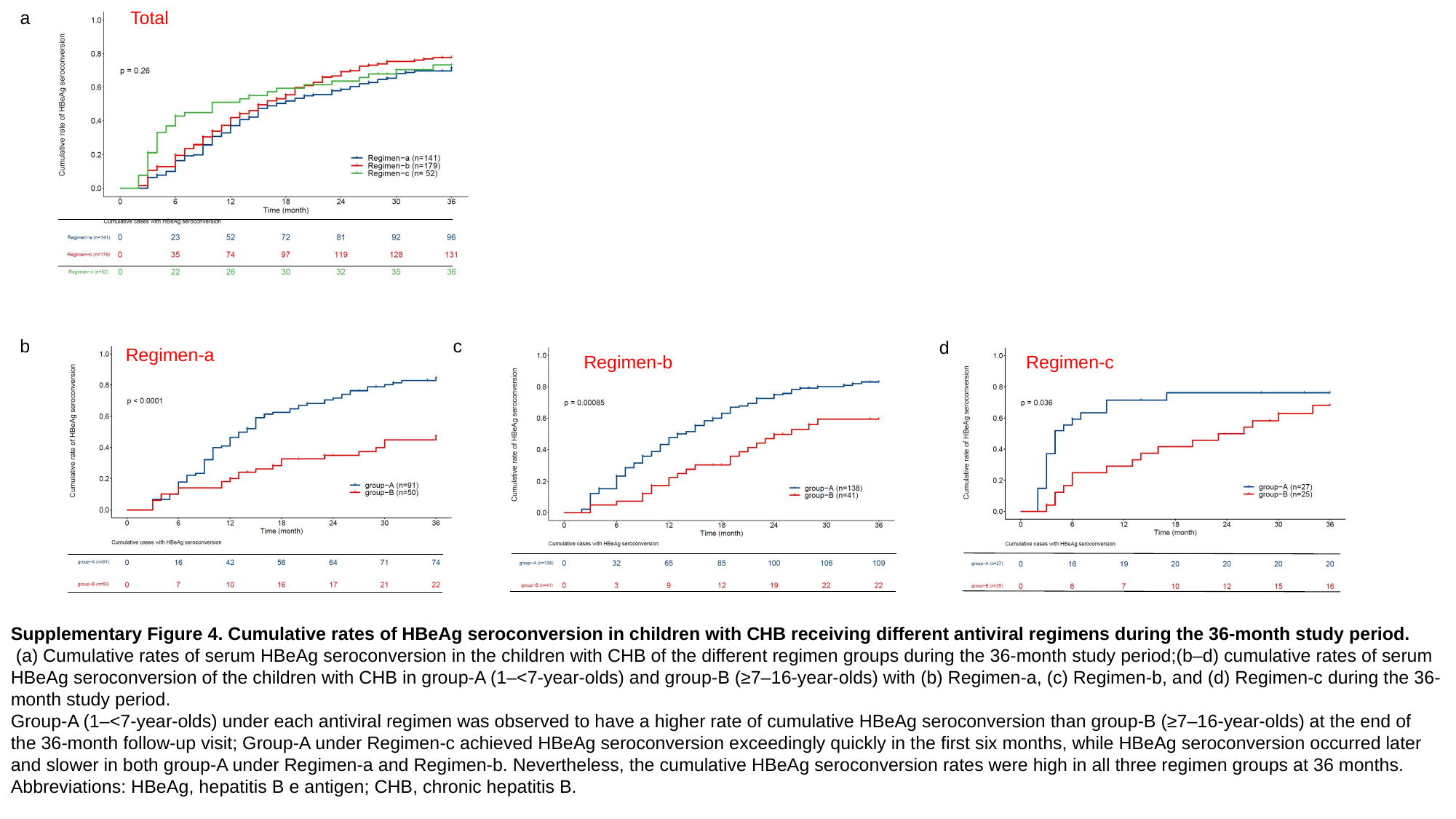

a
Total
b
c
d
Regimen-a
Regimen-b
Regimen-c
Supplementary Figure 4. Cumulative rates of HBeAg seroconversion in children with CHB receiving different antiviral regimens during the 36-month study period.
 (a) Cumulative rates of serum HBeAg seroconversion in the children with CHB of the different regimen groups during the 36-month study period;(b–d) cumulative rates of serum HBeAg seroconversion of the children with CHB in group-A (1–<7-year-olds) and group-B (≥7–16-year-olds) with (b) Regimen-a, (c) Regimen-b, and (d) Regimen-c during the 36-month study period.
Group-A (1–<7-year-olds) under each antiviral regimen was observed to have a higher rate of cumulative HBeAg seroconversion than group-B (≥7–16-year-olds) at the end of the 36-month follow-up visit; Group-A under Regimen-c achieved HBeAg seroconversion exceedingly quickly in the first six months, while HBeAg seroconversion occurred later and slower in both group-A under Regimen-a and Regimen-b. Nevertheless, the cumulative HBeAg seroconversion rates were high in all three regimen groups at 36 months.
Abbreviations: HBeAg, hepatitis B e antigen; CHB, chronic hepatitis B.

## Slide 5
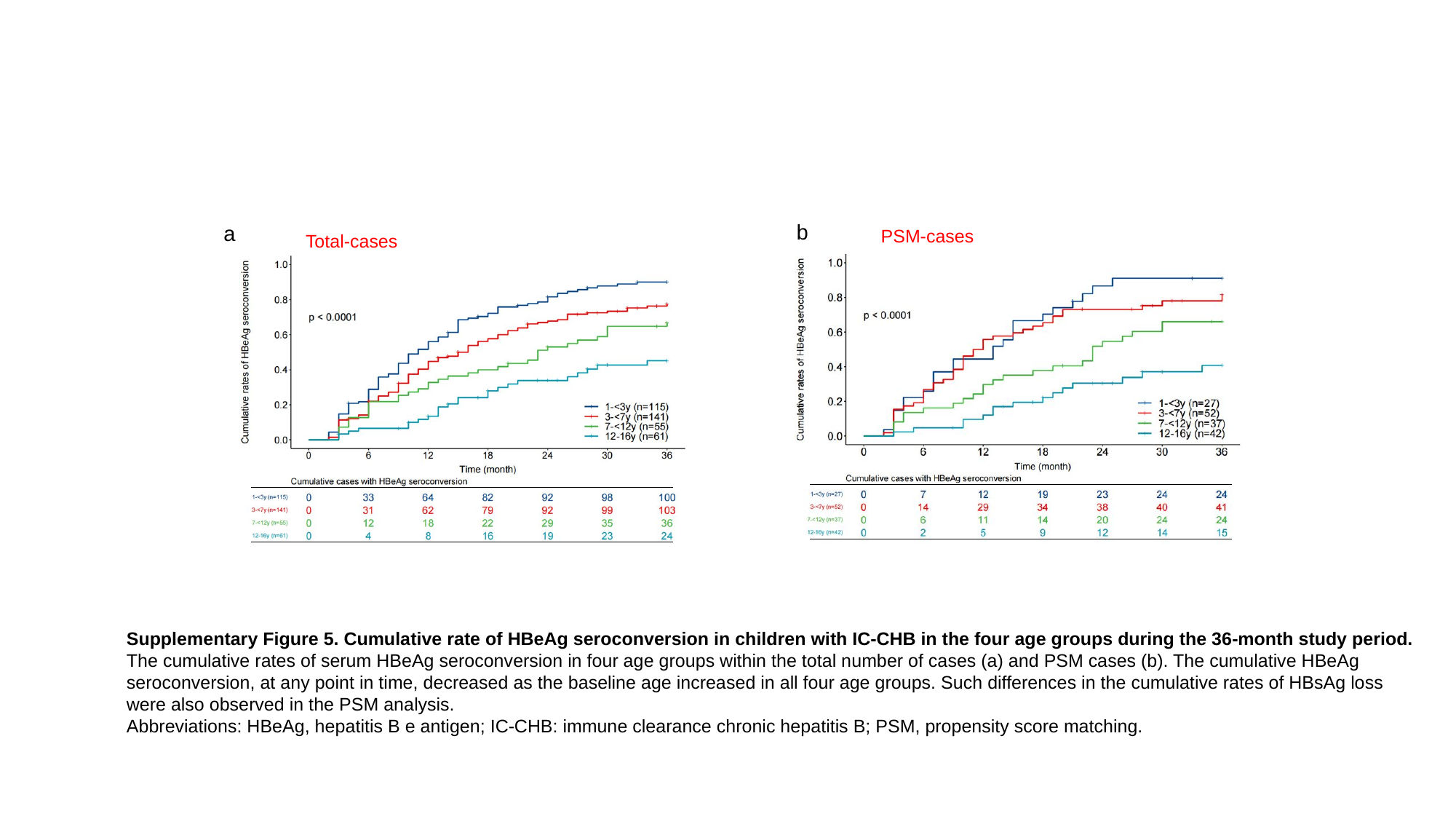

b
a
PSM-cases
Total-cases
Supplementary Figure 5. Cumulative rate of HBeAg seroconversion in children with IC-CHB in the four age groups during the 36-month study period.
The cumulative rates of serum HBeAg seroconversion in four age groups within the total number of cases (a) and PSM cases (b). The cumulative HBeAg seroconversion, at any point in time, decreased as the baseline age increased in all four age groups. Such differences in the cumulative rates of HBsAg loss were also observed in the PSM analysis.
Abbreviations: HBeAg, hepatitis B e antigen; IC-CHB: immune clearance chronic hepatitis B; PSM, propensity score matching.

## Slide 6
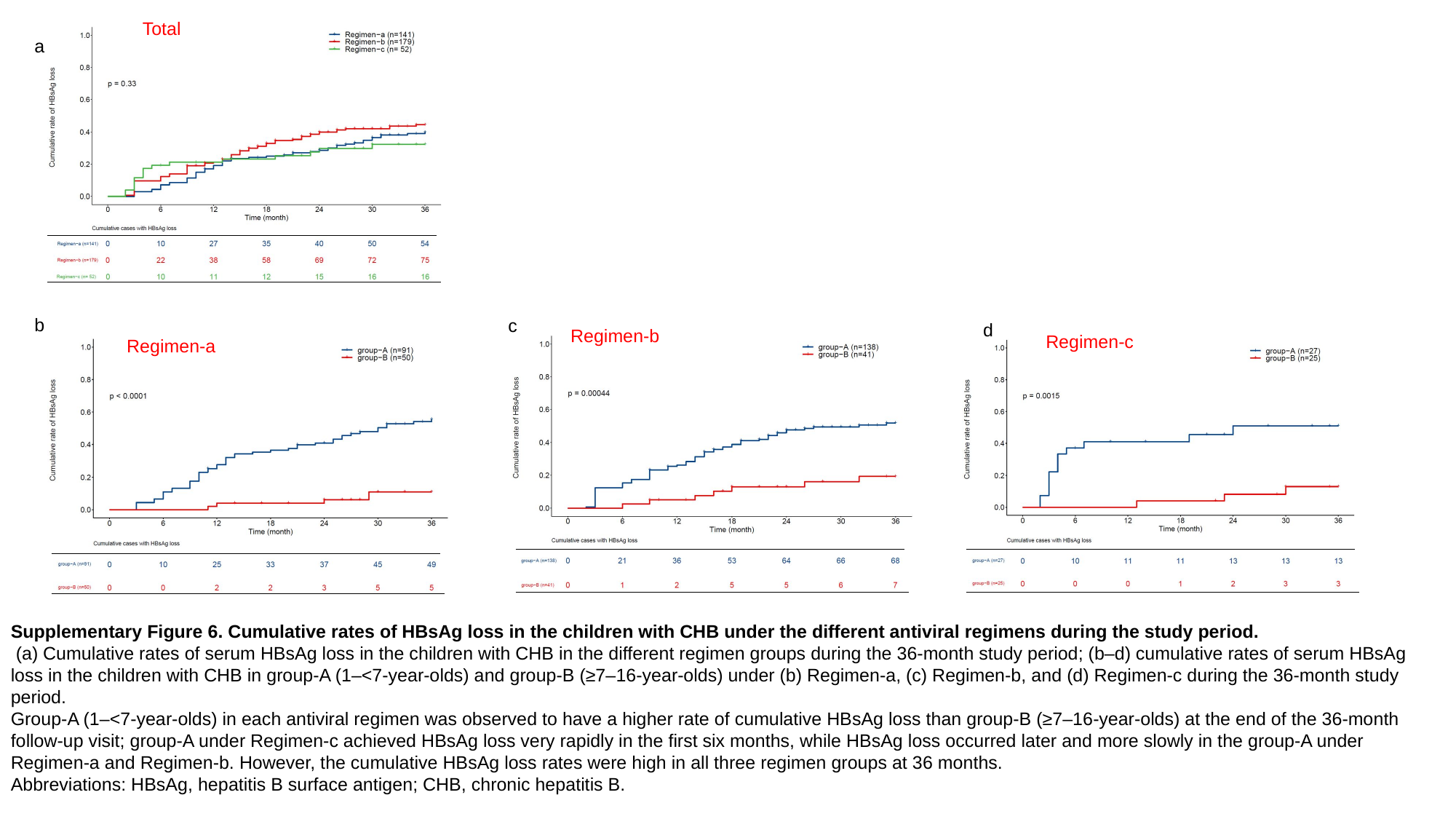

Total
a
b
c
d
Regimen-b
Regimen-c
Regimen-a
Supplementary Figure 6. Cumulative rates of HBsAg loss in the children with CHB under the different antiviral regimens during the study period.
 (a) Cumulative rates of serum HBsAg loss in the children with CHB in the different regimen groups during the 36-month study period; (b–d) cumulative rates of serum HBsAg loss in the children with CHB in group-A (1–<7-year-olds) and group-B (≥7–16-year-olds) under (b) Regimen-a, (c) Regimen-b, and (d) Regimen-c during the 36-month study period.
Group-A (1–<7-year-olds) in each antiviral regimen was observed to have a higher rate of cumulative HBsAg loss than group-B (≥7–16-year-olds) at the end of the 36-month follow-up visit; group-A under Regimen-c achieved HBsAg loss very rapidly in the first six months, while HBsAg loss occurred later and more slowly in the group-A under Regimen-a and Regimen-b. However, the cumulative HBsAg loss rates were high in all three regimen groups at 36 months.
Abbreviations: HBsAg, hepatitis B surface antigen; CHB, chronic hepatitis B.

## Slide 7
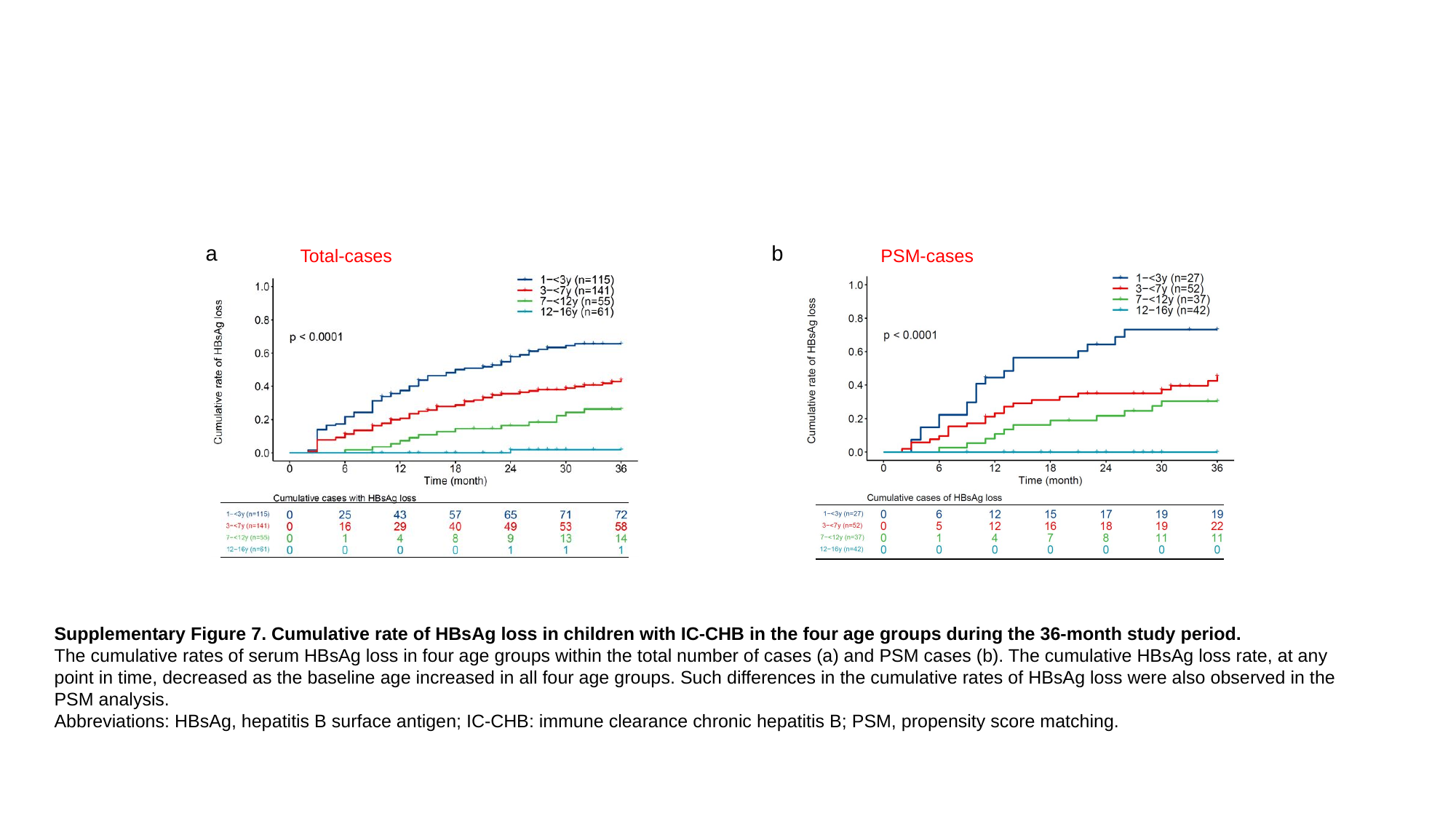

a
b
PSM-cases
Total-cases
Supplementary Figure 7. Cumulative rate of HBsAg loss in children with IC-CHB in the four age groups during the 36-month study period.
The cumulative rates of serum HBsAg loss in four age groups within the total number of cases (a) and PSM cases (b). The cumulative HBsAg loss rate, at any point in time, decreased as the baseline age increased in all four age groups. Such differences in the cumulative rates of HBsAg loss were also observed in the PSM analysis.
Abbreviations: HBsAg, hepatitis B surface antigen; IC-CHB: immune clearance chronic hepatitis B; PSM, propensity score matching.

## Slide 8
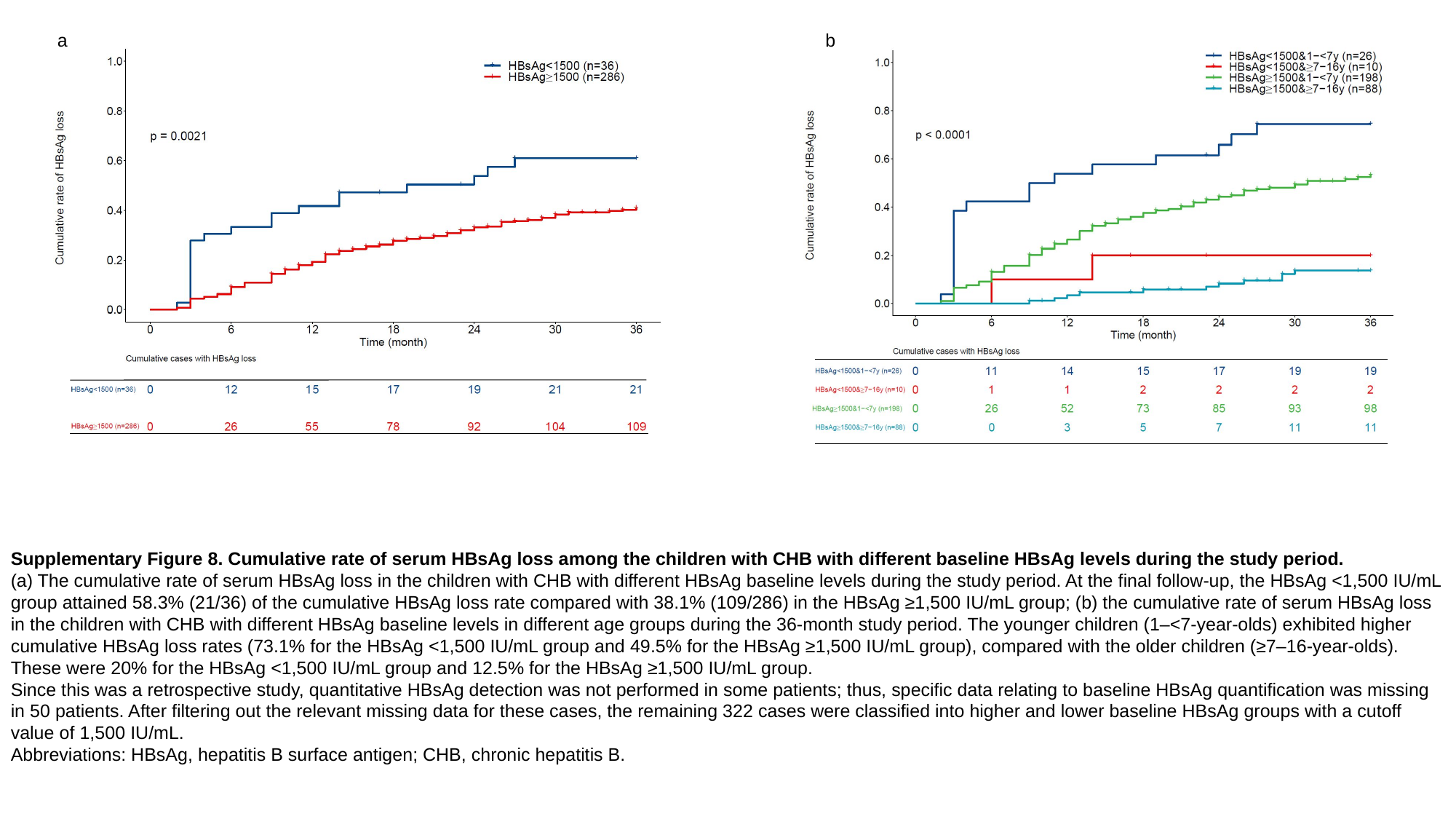

a
b
Supplementary Figure 8. Cumulative rate of serum HBsAg loss among the children with CHB with different baseline HBsAg levels during the study period.
(a) The cumulative rate of serum HBsAg loss in the children with CHB with different HBsAg baseline levels during the study period. At the final follow-up, the HBsAg <1,500 IU/mL group attained 58.3% (21/36) of the cumulative HBsAg loss rate compared with 38.1% (109/286) in the HBsAg ≥1,500 IU/mL group; (b) the cumulative rate of serum HBsAg loss in the children with CHB with different HBsAg baseline levels in different age groups during the 36-month study period. The younger children (1–<7-year-olds) exhibited higher cumulative HBsAg loss rates (73.1% for the HBsAg <1,500 IU/mL group and 49.5% for the HBsAg ≥1,500 IU/mL group), compared with the older children (≥7–16-year-olds). These were 20% for the HBsAg <1,500 IU/mL group and 12.5% for the HBsAg ≥1,500 IU/mL group.
Since this was a retrospective study, quantitative HBsAg detection was not performed in some patients; thus, specific data relating to baseline HBsAg quantification was missing in 50 patients. After filtering out the relevant missing data for these cases, the remaining 322 cases were classified into higher and lower baseline HBsAg groups with a cutoff value of 1,500 IU/mL.
Abbreviations: HBsAg, hepatitis B surface antigen; CHB, chronic hepatitis B.

## Slide 9
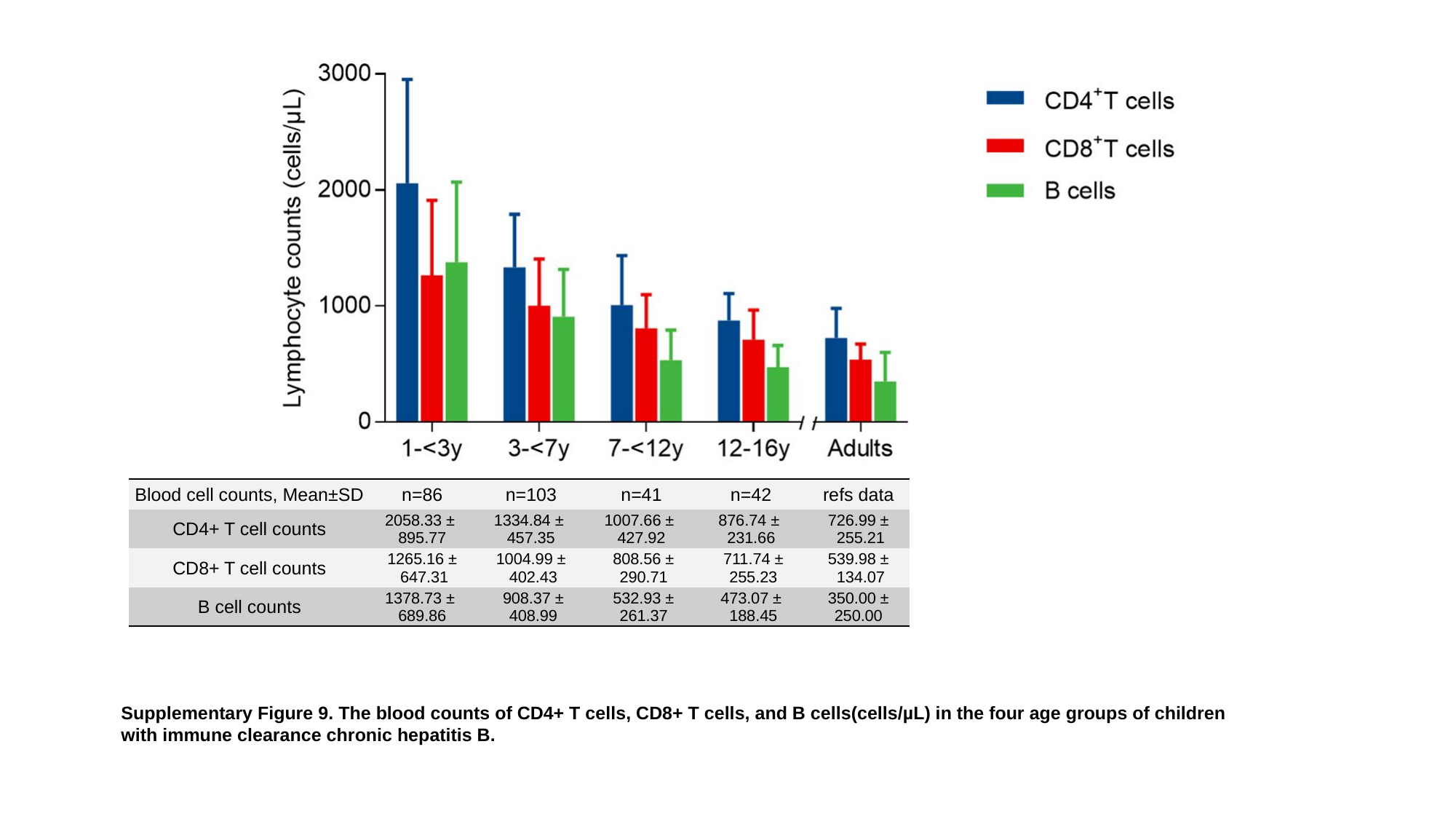

| Blood cell counts, Mean±SD | n=86 | n=103 | n=41 | n=42 | refs data |
| --- | --- | --- | --- | --- | --- |
| CD4+ T cell counts | 2058.33 ± 895.77 | 1334.84 ± 457.35 | 1007.66 ± 427.92 | 876.74 ± 231.66 | 726.99 ± 255.21 |
| CD8+ T cell counts | 1265.16 ± 647.31 | 1004.99 ± 402.43 | 808.56 ± 290.71 | 711.74 ± 255.23 | 539.98 ± 134.07 |
| B cell counts | 1378.73 ± 689.86 | 908.37 ± 408.99 | 532.93 ± 261.37 | 473.07 ± 188.45 | 350.00 ± 250.00 |
Supplementary Figure 9. The blood counts of CD4+ T cells, CD8+ T cells, and B cells(cells/µL) in the four age groups of children with immune clearance chronic hepatitis B.

## Slide 10
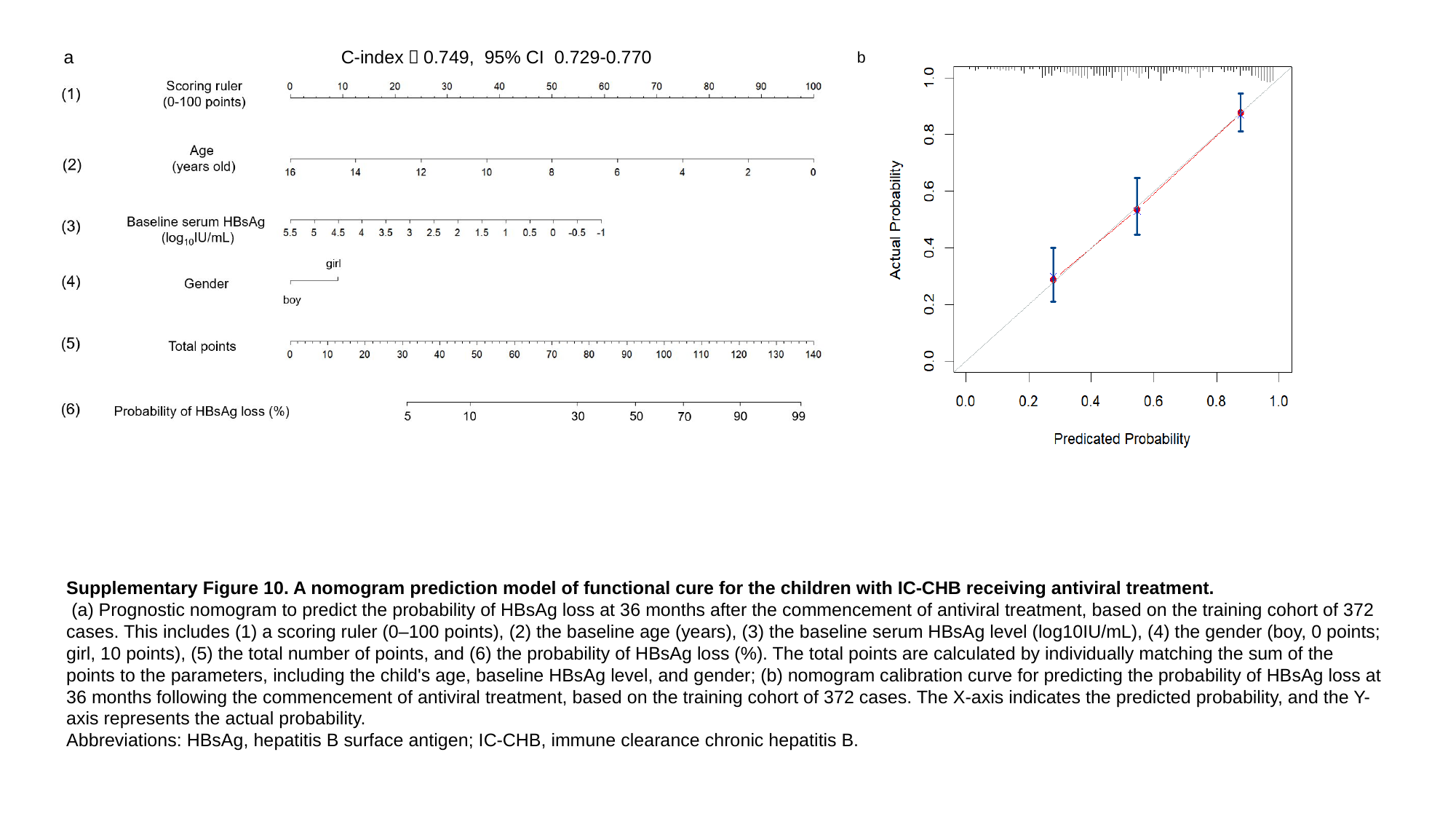

C-index：0.749, 95% CI 0.729-0.770
a
b
Supplementary Figure 10. A nomogram prediction model of functional cure for the children with IC-CHB receiving antiviral treatment.
 (a) Prognostic nomogram to predict the probability of HBsAg loss at 36 months after the commencement of antiviral treatment, based on the training cohort of 372 cases. This includes (1) a scoring ruler (0–100 points), (2) the baseline age (years), (3) the baseline serum HBsAg level (log10IU/mL), (4) the gender (boy, 0 points; girl, 10 points), (5) the total number of points, and (6) the probability of HBsAg loss (%). The total points are calculated by individually matching the sum of the points to the parameters, including the child's age, baseline HBsAg level, and gender; (b) nomogram calibration curve for predicting the probability of HBsAg loss at 36 months following the commencement of antiviral treatment, based on the training cohort of 372 cases. The X-axis indicates the predicted probability, and the Y-axis represents the actual probability.
Abbreviations: HBsAg, hepatitis B surface antigen; IC-CHB, immune clearance chronic hepatitis B.
